# Association between sleep duration and quality with rapid kidney function decline and development of chronic kidney diseases in adults with normal kidney function: The China health and retirement longitudinal study

**DOI:** 10.3389/fpubh.2022.1072238

**Published:** 2023-01-18

**Authors:** Sujuan Xu, Jifu Jin, Qi Dong, Chenjie Gu, Yong Wu, Haibo Zhang, Yingchao Yin, Huiyang Jia, Mingcheng Lei, Junfei Guo, Haixia Xu, Suchi Chang, Feng Zhang, Zhiyong Hou, Liping Zhang

**Affiliations:** ^1^Department of Nephrology, Third Hospital of Hebei Medical University, Shijiazhuang, China; ^2^Department of Orthopaedical Surgery, Third Hospital of Hebei Medical University, Shijiazhuang, China; ^3^Orthopaedic Research Institute of Hebei Province, Hebei Medical University, Shijiazhuang, China; ^4^Department of Cardiovascular Medicine, Shanghai East Hospital, Tongji University School of Medicine, Shanghai, China; ^5^The First Affiliated Hospital of Dalian Medical University, Dalian, China; ^6^Department of Nephrology, Shanghai Ninth People's Hospital, School of Medicine, Shanghai Jiao Tong University, Shanghai, China; ^7^Department of Nephrology, Huashan Hospital, Fudan University, Shanghai, China; ^8^Department of Liver Disease, Affiliated Hospital of Shaanxi University of Chinese Medicine, Xianyang, Shaanxi, China; ^9^Department of Rehabilitation Medicine, Third Hospital of Hebei Medical University, Shijiazhuang, China; ^10^Department of Cardiology, Affiliated Hospital of Nantong University, Jiangsu, China; ^11^Department of Cardiology, Zhongshan Hospital, Shanghai Medical College, Fudan University, Shanghai, China; ^12^Department of Physiology, Hebei Medical University, Shijiazhuang, Hebei, China

**Keywords:** aging, renal function decline, glomerular filtration rate, sleep quality, sleep duration

## Abstract

Research have shown that sleep is associated with renal function. However, the potential effects of sleep duration or quality on kidney function in middle-aged and older Chinese adults with normal kidney function has rarely been studied. Our study aimed to investigate the association of sleep and kidney function in middle-aged and older Chinese adults. Four thousand and eighty six participants with an eGFR ≥60 ml/min/1.73 m^2^ at baseline were enrolled between 2011 and 2015 from the China Health and Retirement Longitudinal Study. Survey questionnaire data were collected from conducted interviews in the 2011. The eGFR was estimated from serum creatinine and/or cystatin C using the Chronic Kidney Disease Epidemiology Collaboration equations (CKD-EPI). The primary outcome was defined as rapid kidney function decline. Secondary outcome was defined as rapid kidney function decline with clinical eGFR of <60 ml/min/1.73 m^2^ at the exit visit. The associations between sleep duration, sleep quality and renal function decline or chronic kidney disease (CKD) were assessed based with logistic regression model. Our results showed that 244 (6.0%) participants developed rapid decline in kidney function, while 102 (2.5%) developed CKD. In addition, participants who had 3–7 days of poor sleep quality per week had higher risks of CKD development (OR 1.86, 95% CI 1.24–2.80). However, compared with those who had 6–8 h of night-time sleep, no significantly higher risks of rapid decline in kidney function was found among those who had <6 h or >8 h of night time sleep after adjustments for demographic, clinical, or psychosocial covariates. Furthermore, daytime nap did not present significant risk in both rapid eGFR decline or CKD development. In conclusion, sleep quality was significantly associated with the development of CKD in middle-aged and older Chinese adults with normal kidney function.

## Introduction

Chronic kidney disease (CKD) is a detrimental public health issue with an increasing prevalence and complications worldwide (1). In 2012, the overall prevalence of CKD was 11% in Chinese adults (2, 3). As CKD is closely linked to the increased risk of various disease, such as diabetes mellitus (DM), hypertension, metabolic disorders, and cardiovascular disease (2), early identification and intervention of modifiable lifestyle-related risk factors for CKD are recognized as an effective option for preventing the development of this disease (4).

Sleep is an indispensable element for optimal heath and quality of life. In recent years, accelerated aging in China raises serious concerns for middle-aged and older persons, where the circadian mechanisms increasingly become less efficient. Consequently, older people tend to sleep less and have poor sleep quality, which may lead to multiple chronic diseases, such as depression, headache, memory loss, CKD, obesity, DM, and hypertension (5–9). Epidemiological studies demonstrated that prevalence of sleep disturbances in CKD was ~80% (10), and sleep duration and quality were modifiable risk factors that could effectively prevent CKD. Mechanistically, this relationship is associated with sympathetic overreaction, circadian rhythm and metabolic disorders (11, 12). Several studies showed that inadequate duration and poor quality of sleep were increasingly associated with decline of kidney function and development of proteinuria (13–15). Furthermore, a recent study revealed that short or long sleep duration were related to the increased risk of CKD when compared with intermediate sleep duration (8). In contrary, previous meta-analysis indicated that short sleep duration was closely related to proteinuria rather than CKD development (16). Inconsistent findings such as these indicate that further studies focused on the association between sleep disturbances and CKD development needs to be evaluated.

To address the above inconsistencies, this study explored whether sleep duration, quality were deleterious factors for rapid decline of renal function and the development of CKD in middle-aged and older Chinese adults within The China Health and Retirement Longitudinal Study (CHARLS) database, a nationally representative, longitudinal cohort with the measurements of serum creatinine and cystatin C.

## Materials and methods

### Study participants and design

The China Health and Retirement Longitudinal Study (CHARLS) (17) was a project implemented using a multistage, stratified and proportionate-to-size sampling method. CHARLS included 17,708 participants from 150 counties and 450 villages within 28 provinces in mainland China. The baseline survey was carried out from June 2011 to March 2012. The detailed design and methods on the demographic, lifestyle factors, clinical or biochemical measurements and blood samples in the study were reported previously (17, 18). CHARLS data, which were collected from representative participants of 45 years old and above from among the Chinese population, aimed to establish a higher quality database.

The CHARLS prospective longitudinal cohort included data collected from two time-points (2011, 2015). The exclusion criteria for this study were as follow: participants whose ages were under 45 years old; participants with missing information of baseline sleep duration, baseline sleep quality, baseline kidney functions, exit kidney outcomes and related information such as demographic, lifestyle factors, clinical or biochemical measurements. Based on these criteria, data from 7,761 participants were excluded, and a total of 4,086 participants with eGFR ≥60 ml/min 1.73 m^2^ at baseline were included (Supplementary Figure 1).

Ethical approval of CHARLS was authorized by the Biomedical Ethics Review Committee of Peking University (IRB00001052–11015) (17). All participants have signed and provided written informed consent before participating in the survey. Information on the materials for this study are available on the CHARLS project website.

### Assessment of sleep duration and quality

Sleep duration and quality were collected from the baseline survey carried out in 2011 (17). The standardized question used was, “How many hours of sleep did you get per night (average hours per night-time sleep) during the past month?” The night-time sleep duration were stratified into three categories: short (< 6 h/night), intermediate (6–8 h/night), and long (>8 h/night) (8, 14, 19). We selected < 6 h as the definition for short sleep in the analysis to include those who have short sleeping duration despite self-reported sleep duration.

Daytime nap duration was self-reported by participants with the question: “How long did a nap last on average during the past month?.” Consistent with previous literatures (20, 21), participants were categorized into four groups: non-nappers (0 h), short nappers (< 0.5 h), moderate nappers (0.5–1.5 h) and extended nappers (>1.5 h).

Sleep quality was assessed by “How many days of restless sleep in a week?.” We classified sleep quality into two categories: rarely or a little (0–2 days/week); and occasionally, most or all of the time (3–7 days/week).

### Assessment of kidney function

The estimated glomerular filtration rate (eGFR) was calculated from serum creatinine and/or cystatin C using the Chronic Kidney Disease Epidemiology Collaboration equations (CKD-EPI)(22). The eGFR_cr − cys_ was calculated using the CKD-EPI creatinine-cystatin C equation:


$$\begin{array}{l} {\text{eGFR}}_{\text{cr-cys}}\;=\;135\times \text{min}{\left(\text{Scr}/\text{k},\text{1}\right)}^{\alpha }\times \text{max}{\left(\text{Scr}/\text{k},1\right)}^{-0.601}\\ \;\times \text{ min}{\left(\text{Scys}/0.8,1\right)}^{-0.375}\times \text{max}{\left(\text{Scys}/0.8,1\right)}^{-0.711}\\ \;\times \;0.995^{\text{age}}[\times 1.08\text{ if black}]\times 0.969[\text{if}\;\text{female}]; \end{array}$$

where Scr is serum creatinine in mg/dl, Scys is serum cystatin C in mg/l, k is 0.7 for females and 0.9 for males, and α is −0.248 for females and −0.207 for males.

The eGFR_cr_ was calculated using the CKD-EPI creatinine equation: eGFR_cr_ = 141 × min (Scr /k, 1) ^α^ × max (Scr /k, 1) ^−1.209^ × 0.993^age^ × 1.159 [if black] × 1.018 [if female];

Where, k is 0.7 for females and 0.9 for males, α is −0.329 for females and −0.411 for males.

The eGFR_cys_ was calculated by CKD-EPI cystatin C equation: eGFR_cys_ = 133 × min (Scys /0.8, 1) ^−0.499^ × max (Scys /0.8, 1) ^−1.328^ × 0.996^age^ × 0.932 [if female].

### Study outcomes

The primary outcome was rapid kidney function decline, which was defined as an annualized decline in eGFR_cr − cys_ or eGFR_cr_ or eGFR_cys_ of ≥5 ml/min/ 1.73 m^2^ (23). Annualized eGFR decline was estimated by the formula of (eGFR at baseline - eGFR at exit)/follow-up time (4 years).

The secondary outcome was the progression to CKD, which was defined by an annualized decline in eGFR of ≥5 ml/min/1.73 m^2^ and final eGFR < 60 ml/min/1.73 m^2^ at exit.

### Assessment of covariates

Participants voluntarily provided their demographic information, health related data and laboratory results at baseline from the questionnaires in CHARLS survey. Marital status was categorized into two groups: unmarried and married, unmarried included never married, separated, divorced and widowed. Educational level was categorized into four groups: illiterate, literate, primary school and middle school or above. Blood pressure, height and weight were measured with calibrated equipment. Body mass index (BMI) was calculated as weight/height^2^. Health-related factors, such as smoking, drinking, diabetes and heart disease were self-reported. Diabetes was defined as random glucose ≥200 mg/dl, fasting glucose ≥126 mg/dl, hemoglobin A1c ≥7% and physician-diagnosed diabetes or the use of hypoglycemic drugs.

### Statistical analyses

Baseline characteristics of the population are shown as means ± standard deviations (SD) for continuous variables and as numbers and proportions for categorical variables in the categories of sleep duration or quality. One-way ANOVA analysis of variance, student's *t*-test or chi-squared tests were used to compare the characteristics of participants based on the categories of sleep duration or quality.

Univariate and multivariable logistic regression models were used to investigate the association between sleep duration, quality and kidney outcomes with adjustments for baseline eGFR in model 1 and model 2. In addition, adjusted covariates in model 2 included age, sex, BMI, smoking status, living residence, blood pressure, self-reported heart disease, glucose, total cholesterol, triglycerides, high-density lipoprotein (HDL) cholesterol and uric acid. These were presented as adjusted odds-ratios (ORs) with 95% confidence interval (95% CI).

Furthermore, potential modifications of the relationship among sleep duration, quality and rapid kidney function decline were investigated for the following variables: age, sex, BMI, smoking status, living residence, marital status, educational level, diabetes, total cholesterol, uric acid and high-sensitivity C-reactive protein (CRP) *via* stratified analyses and interaction testing. These variables were either suspected or traditional risk factors for kidney function decline.

IBM SPSS version 23.0 (IBM Corporation, Armonk, NY, USA) was used for statistical analyses in our study. *P* < 0.05 was considered as statistically significant in all analyses.

## Results

### Participants baseline characteristics

Supplementary Figure 1 illustrated the baseline characteristics of participants, where a total of 4,086 analyzed participants with eGFR_cr − cys_ ≥60 ml/min/ 1.73 m^2^ at baseline, their sleep duration and quality from CHARLS were included. The mean age of the included participants was 58.9 ± 8.7 years, 1,755 (43.0%) were male, which is shown in Supplemental Table 1, the mean eGFR_cr − cys_ was 86.2 ± 14.4 ml/min/1.73 m^2^ at baseline.

Compared with participants of 6–8 h of night-time sleep, majority of those in the category of < 6 h or >8 h of night-time sleep were unmarried, farmers, and less educated. Additionally, those in the category of < 6 h of night time sleep showed a trend toward higher HDL cholesterol levels and lower baseline eGFR (Table 1). According to the daytime nap duration (Table 2), participants in the extended nappers group were mostly males, with a trend toward lower HDL cholesterol levels. Table 3 showed the baseline characteristics of participants in sleep quality (0–2 or 3–7 days of poor sleep quality). Participants with poor sleep quality were older and less educated, mostly females, non-smokers and non-drinkers. There were also a higher percentage of heart disease observed in these participants, presenting with lower BMI and uric acid in serum.

**Table 1:** Participants (*n* = 4,086) characteristics by night sleep duration categories.

**Sleep duration per night in hours**					***P*-value**
	**Overall**	<**6 h**	**6–8 h**	>**8 h**	
*N*	4,086	1,228	2,531	327	
Sleep duration, hour	6.3 ± 1.9	4.1 ± 1.1	7.0 ± 0.8	9.6 ± 0.8	<**0.001**
Age, year	58.9 ± 8.7	60.7 ± 8.7	58.0 ± 8.5	59.2 ± 9.3	<**0.001**
Male, no. (%)	1,755 (43.0)	495 (40.0)	1,129 (51.0)	131 (40.1)	**0.024**
Body mass index, kg/m^2^	23.7 ± 3.9	23.2 ± 3.9	23.9 ± 3.8	23.7 ± 4.3	<**0.001**
Rural residence, no. (%)	3,419 (83.7)	1,050 (85.5)	2,075 (82.0)	294 (90.0)	<**0.001**
Married, no. (%)	3,618 (88.5)	1,047 (85.2)	2,297 (90.8)	274 (83.8)	<**0.001**
Smoking status, no. (%)	1,170 (28.6)	359 (29.2)	732 (28.9)	79 (24.1)	0.172
Drinking status, no. (%)	1,282 (31.4)	363 (29.6)	820 (32.4)	99 (30.3)	0.193
Systolic BP, mmHg	130.2 ± 21.4	130.0 ± 21.6	130.0 ± 21.1	131.4 ± 22.2	0.559
Diastolic BP, mmHg	75.6 ± 10.9	75.8 ± 11.0	75.5 ± 10.7	75.8 ± 11.2	0.725
Diabetes, no. (%)	326 (8.0)	102 (8.3)	192 (7.6)	32 (9.8)	0.495
Self-reported heart disease, no. (%)	500 (12.2)	180 (14.6)	293 (11.5)	27 (8.3)	**0.002**
**Education, no. (%)**					<**0.001**
Illiteracy	1,207 (29.5)	442 (36.0)	649 (25.6)	116 (35.5)	
Literate	779 (19.1)	275 (22.4)	448 (17.7)	56 (17.1)	
Primary school	936 (22.9)	273 (22.2)	577 (22.8)	86 (26.3)	
Middle school or above	1,164 (29.5)	238 (19.4)	858 (33.9)	68 (20.8)	
**Laboratory results**					
Total cholesterol, mg/dl	193.5 ± 37.9	194.1 ± 38.0	192.9 ± 37.6	196.2 ± 39.9	0.252
Triglycerides, mg/dl	131.7 ± 90.8	131.5 ± 93.5	130.7 ± 88.7	139.7 ± 96.7	0.243
HDL cholesterol, mg/dl	51.0 ± 15.1	52.4 ± 15.9	50.5 ± 14.7	50.1 ± 15.3	**< 0.001**
LDL cholesterol, mg/dl	116.6 ± 34.9	115.5 ± 34.7	117.0 ± 34.9	118.0 ± 35.4	0.397
Glucose, mg/dl	109.6 ± 34.2	109.3 ± 33.1	109.6 ± 34.5	110.7 ± 37.0	0.807
Hemoglobin A1c, %	5.3 ± 0.8	5.2 ± 0.7	5.3 ± 0.8	5.3 ± 1.0	**0.018**
eGFRcr-cys, ml/min per 1.73 m^2^	86.2 ± 14.4	84.7 ± 13.9	86.6 ± 14.5	88.4 ± 15.4	**< 0.001**
Uric acid, mg/dl	4.3 ± 1.1	4.3 ± 1.1	4.3 ± 1.2	4.2 ± 1.1	0.177
High-sensitivity CRP, mg/L	2.3 ± 5.5	2.4 ± 5.9	2.2 ± 5.5	1.8 ± 2.8	0.164

Variables are presented as mean ± standard deviation or numbers (percentages).

*p*-values were calculated with one-way ANOVA analysis for continuous variables and chi-squared test for categorical variables. BP, blood pressure; HDL Cholesterol, high density lipoprotein cholesterol; LDL Cholesterol, low density lipoprotein cholesterol; eGFRcr-cys, eGFR on the basis of a combination of serum creatinine and cystatin C; CRP, C-reactive protein. Bold values was considered as statistically significant.

**Table 2:** Participants (*n* = 4,086) characteristics by daytime nap categories.

	**Overall**	**Daytime nap time**				***P*-value**
		**Non nappers**	**Short nappers**	**Moderate nappers**	**Extended nappers**	
*N*	4,086	1,882	388	1,244	572	
Nap time, %	100.0	46.1	9.5	30.4	14.0	<**0.001**
Age, year	58.9 ± 8.7	58.6 ± 8.5	58.8 ± 8.3	59.3 ± 9.0	9.1	0.141
Male, no. (%)	1,755 (43.0)	667 (35.4)	190 (49.0)	604 (48.4)	294 (51.4)	**< 0.001**
Body mass index, kg/m^2^	23.7 ± 3.9	23.5 ± 4.1	23.9 ± 3.7	23.7 ± 3.8	23.9 ± 3.7	**0.044**
Rural residence, no. (%)	3419 (83.7)	1609 (86.4)	311 (80.2)	1018 (81.8)	481 (84.1)	**0.001**
Married, no. (%)	3,618 (88.5)	1,663 (88.4)	354 (91.2)	1,100 (88.4)	501 (87.6)	0.338
Smoking status, no. (%)	1,170 (28.6)	501 (26.6)	104 (26.8)	358 (28.8)	204 (35.7)	**0.001**
Drinking status, no. (%)	1,282 (31.4)	511 (27.2)	139 (35.8)	418 (33.6)	214 (37.4)	**0.001**
Systolic BP, mmHg	130.2 ± 21.4	127.4 ± 21.5	129.0 ± 19.2	132.4 ± 20.6	135.2 ± 22.4	**< 0.001**
Diastolic BP, mmHg	75.6 ± 10.9	74.2 ± 11.0	75.8 ± 10.1	76.7 ± 10.3	77.7 ± 11.5	<**0.001**
Diabetes, no. (%)	326 (8.0)	133 (7.1)	33 (8.5)	115 (9.2)	45 (7.9)	0.172
Self-reported heart disease, no. (%)	500 (12.2)	209 (11.1)	57 (14.7)	169 (13.6)	65 (11.4)	0.074
**Education, no. (%)**						<**0.001**
Illiteracy	1,207 (29.5)	633 (33.8)	90 (23.2)	341 (27.4)	143 (25.0)	
Literate	779 (19.1)	365 (19.5)	59 (15.2)	244 (19.6)	111 (19.4)	
Primary school	936 (22.9)	403 (21.5)	100 (25.8)	290 (23.3)	143 (25.0)	
Middle school or above	1,164 (29.5)	471 (25.2)	139 (35.8)	369 (29.7)	175 (30.6)	
**Laboratory results**						
Total cholesterol, mg/dl	193.5 ± 37.9	193.2 ± 37.0	196.4 ± 41.7	193.5 ± 38.0	192.7 ± 38.2	0.440
Triglycerides, mg/dl	131.7 ± 90.8	128.9 ± 88.1	130.4 ± 81.4	132.5 ± 94.7	139.8 ± 96.7	0.094
HDL cholesterol, mg/dl	51.0 ± 15.1	52.3 ± 15.2	50.0 ± 14.0	50.6 ± 15.8	48.7 ± 13.8	**< 0.001**
LDL cholesterol, mg/dl	116.6 ± 34.9	115.9 ± 34.6	121.0 ± 37.6	116.5± 34.0	116.3 ± 32.9	0.073
Glucose, mg/dl	109.6 ± 34.2	108.0 ± 32.8	109.5 ± 32.3	111.1 ± 37.4	111.6 ± 37.0	**0.037**
Hemoglobin A1c, %	5.3 ± 0.8	5.2 ± 0.8	5.3 ± 0.8	5.3 ± 0.8	5.3 ± 0.8	0.133
eGFRcr-cys, ml/min per 1.73 m^2^	86.2 ± 14.4	86.6 ± 14.3	85.2 ± 14.2	85.6 ± 15.4	86.5 ± 14.8	0.110
Uric acid, mg/dl	4.3 ± 1.1	4.2 ± 1.1	4.3 ± 1.1	4.2 ± 1.2	4.2 ± 1.2	**0.020**
High-sensitivity CRP, mg/L	2.3 ± 5.5	2.1 ± 5.0	2.3 ± 5.1	2.2 ± 5.6	2.9 ± 6.9	**0.018**

Variables are presented as mean ± standard deviation or numbers (percentages).

*p*-values were calculated with one-way ANOVA analysis for continuous variables and chi-squared test for categorical variables. BP, blood pressure; HDL, Cholesterol, high density lipoprotein cholesterol; LDL, Cholesterol, low density lipoprotein cholesterol; eGFRcr-cys, eGFR on the basis of a combination of serum creatinine and cystatin C; CRP, C-reactive protein. Bold values was considered as statistically significant.

**Table 3:** Participants (*n* = 4,086) characteristics by poor sleep quality categories.

	**Overall**	**Days of poor sleep quality in the past week**		***P*-value**
		**0–2 d**	**3–7 d**	
*N*	4,086	2,630	1,456	
Poor sleep quality, day	2.1 ± 1.2	1.3 ± 0.4	3.6 ± 0.5	<**0.001**
Age, year	58.9 ± 8.7	58.6 ± 8.7	59.7 ± 8.7	<**0.001**
Male, no. (%)	1755 (43.0)	1244 (47.3)	511 (35.1)	<**0.001**
Body mass index, kg/m^2^	23.7 ± 3.9	23.8 ± 3.9	23.4 ± 3.8	<**0.001**
Rural residence, no. (%)	3,419 (83.7)	2,174 (82.7)	1,245 (84.0)	**0.001**
Married, no. (%)	3,618 (88.5)	2,355 (89.5)	1,263 (86.7)	**0.007**
Smoking status, no. (%)	1,170 (28.6)	800 (28.4)	367 (25.2)	**< 0.001**
Drinking status, no. (%)	1,282 (31.4)	363 (33.3)	820 (28.0)	**< 0.001**
Systolic BP, mmHg	130.2 ± 21.4	130.1 ± 21.1	130.1 ± 21.8	0.990
Diastolic BP, mmHg	75.6 ± 10.9	75.6 ± 10.8	75.6 ± 11.0	0.971
Diabetes, no. (%) Self-	326 (8.0)	200 (7.6)	126 (8.7)	0.236
Reported heart disease, no. (%)	500 (12.2)	278 (10.6)	222 (15.2)	**< 0.001**
**Education, no. (%)**				< 0.001
Illiteracy	1207 (29.5)	683 (26.0)	524 (36.2)	
Literate	779 (19.1)	484 (18.4)	295 (20.4)	
Primary school	936 (22.9)	613 (23.3)	323 (22.3)	
Middle school or above	1,164 (29.5)	850 (32.3)	858 (21.0)	
**Laboratory results**				
Total cholesterol, mg/dl	193.5 ± 37.9	193.4 ± 38.4	193.8 ± 37.6	0.757
Triglycerides, mg/dl	131.7 ± 90.8	132.4 ± 91.1	130.4 ± 90.2	0.496
HDL cholesterol, mg/dl	51.0 ± 15.1	50.6 ± 15.1	51.8 ± 15.2	0.015
LDL cholesterol, mg/dl	116.6 ± 34.9	116.8 ± 35.4	116.3 ± 33.9	0.655
Glucose, mg/dl	109.6 ± 34.2	109.6 ± 33.8	109.4 ± 35.1	0.852
Hemoglobin A1c, %	5.3 ± 0.8	5.3 ± 0.8	5.3 ± 0.8	0.789
eGFRcr-cys, ml/min per 1.73 m^2^	86.2 ± 14.4	86.3 ± 14.6	86.0 ± 14.1	0.458
Uric acid, mg/dl High-	4.3 ± 1.1	4.3 ± 1.2	4.2 ± 1.1	<**0.001**
sensitivity CRP, mg/L	2.3 ± 5.5	2.3 ± 5.8	2.2 ± 4.8	0.661

Variables are presented as mean ± standard deviation or numbers (percentages).

*p*-values were calculated with two-sample t tests for continuous variables and chi-squared test for categorical variables.

BP, blood pressure; HDL Cholesterol, high density lipoprotein cholesterol; LDL Cholesterol, low density lipoprotein cholesterol; eGFRcr-cys, eGFR on the basis of a combination of serum creatinine and cystatin C; CRP, C-reactive protein. Bold values was considered as statistically significant.

Supplementary Table 1 showed the baseline characteristics of excluded participants. Compared with those who were included, participants excluded were more commonly urban men, smokers, non-drinkers, more educated and had higher triglycerides and uric acid.

### Association between sleep duration, quality, and study outcomes

Based on data followed up for a median of 4 years, 244 (6.0%) participants developed rapid declines in kidney function, and 102 (2.5%) progressed to CKD.

In the demographic, clinical, or psychosocial covariates adjusted model (Model 2), participants in the category of < 6 h or >8 h of night-time sleep were similar in their risks for both rapid eGFR decline and CKD development compared to those with 6–8 h of nighttime sleep (Supplementary Table 2). The effects of day time nap on kidney function is shown in Supplementary Table 3. Non-nappers, short-time nappers and extended-time nappers were similar in their risks for both rapid eGFR decline and CKD development compared with moderate nappers in this analysis. When sleep qualities were assessed, the adjusted ORs for participants with 3–7 days of poor sleep quality who developed CKD was 1.86 (95% CI, 1.24 to 2.80) compared with those with 0–2 days of poor sleep quality (Table 4).

**Table 4:** The association between sleep quality and CKD outcomes.

**CKD outcomes**	**Restless sleep (days/week)**	**Events/*N* (%)**	**Odds ratio (95% CI)** ***P*****-value**	
			**Model 1**	**Model 2**
**Rapid decline in kidney function**				
	0–2	148/2,630 (5.6%)	Reference	Reference
	3–7	96/1,456 (6.6%)	1.24 (0.95–1.63) 0.119	1.31 (0.98–1.76) 0.068
**Progression to CKD**				
	0–2	51/2,630 (1.9%)	Reference	Reference
	3–7	51/1,456 (3.5%)	1.83 (1.24–2.72) **0.003**	1.86 (1.24–2.80) **0.003**

Model 1 was adjusted for eGFRcr-cys at baseline.

Model 2 was adjusted for age, gender, body mass index, smoking status, rural residence, systolic BP, diastolic BP, self-reported heart disease, glucose, total cholesterol, triglycerides, HDL cholesterol, eGFRcr-cys, uric acid.

BP, blood pressure; HDL Cholesterol, high density lipoprotein cholesterol; eGFRcr-cys, eGFR on the basis of a combination of serum creatinine and cystatin C; CKD, chronic kidney disease; CI, confidence interval. Bold values was considered as statistically significant.

The associations of sleep duration, quality and the kidney function were further investigated in Supplementary Tables 4–9. Similar trends were observed in the association between sleep duration, quality, kidney primary and secondary outcomes defined by eGFR_cr_ (Supplementary Tables 4–6) or eGFR_cys_ (Supplementary Tables 7–9), though some of the comparisons were not statistically significant.

### Stratified analyses by potential effect modifiers

Stratified logistic regression analysis for associations between sleep quality and rapid eGFR_cr − cys_ decline through the adjustment of several variables are shown in Table 5 and Supplementary Tables 10–13.

**Table 5:** Stratified analyses for the association between days of poor sleep quality and rapid kidney function decline.

**Subgroup**	***N***	**Days of poor sleep quality**		**OR(95%CI)**	***P* for interaction**
		**0–2 d**	**3–7 d**		
**Age, years**					0.18
< 65	3,020	110 (5.6)	59 (5.6)	1.15 (0.80–1.64)	
≥65	1,066	38 (5.8)	37 (9.1)	1.70 (1.00–2.91)	
**Body mass index, kg/m** ^**2**^					0.46
< 24	2,370	76 (5.1)	55 (6.2)	1.28 (0.86–1.90)	
≥24	1,708	72 (6.3)	41 (7.3)	1.45 (0.95–2.23)	
**Sex**					0.01
Male	1,755	75 (6.0)	41 (8.0)	1.32 (0.85–2.03)	
Female	2,331	73 (5.3)	55 (5.8)	1.26 (0.85–1.88)	
**Smoking status**					0.03
No	2,916	99 (5.4)	72 (6.6)	1.43 (1.01–2.03)	
Yes	1,170	49 (6.1)	24 (6.5)	1.06 (0.61–1.84)	
**Drinking status**					0.69
No	2,804	100 (5.7)	68 (6.5)	1.35 (0.95–1.92)	
Yes	1,282	48 (5.5)	28 (6.9)	1.33 (0.78–2.27)	
**Marital status**					0.78
Married	3,618	126 (5.4)	77 (6.1)	1.29 (0.93–1.78)	
Unmarried	468	22 (8.0)	19 (9.8)	1.60 (0.74–3.45)	
**Diabetes**					0.09
No	3,760	129 (5.3)	79 (5.9)	1.23 (0.89–1.69)	
Yes	326	19 (9.5)	17 (13.5)	2.16 (0.93–5.03)	
**Educational level**					0.25
Illiteracy or literate	1,986	65 (5.6)	58 (7.1)	1.50 (1.01–2.24)	
Primary school or above	2,100	83 (5.7)	38 (6.0)	1.13 (0.73–1.75)	
**Total cholesterol, mg/dl**					0.06
< 200	2,438	78 (4.9)	51 (5.9)	1.36 (0.92–2.01)	
≥200	1,648	70 (6.6)	45 (7.6)	1.26 (0.81–1.96)	
**Uric acid, mg/dl**					0.15
< 4.2	2,116	69 (5.0)	39 (4.8)	1.07 (0.69–1.65)	
≥4.2	1,970	79 (2.5)	57 (8.8)	1.52 (1.02–2.27)	
**High-sensitivity CRP, mg/L**					0.35
< 1.0	2,079	67 (5.0)	42 (5.6)	1.28 (0.83–1.98)	
≥1.0	2,007	81 (6.2)	54 (7.6)	1.33 (0.89–1.99)	

The model was adjusted, if not stratified, for age, gender, body mass index, smoking status, rural residence, systolic BP, diastolic BP, self-reported heart disease, glucose, total cholesterol, triglycerides, HDL cholesterol, eGFRcr-cys, uric acid.

BP, blood pressure; HDL Cholesterol, high density lipoprotein cholesterol; eGFRcr-cys, eGFR on the basis of a combination of serum creatinine and cystatin C; CKD, chronic kidney disease; CI, confidence interval.

None of the variables such as age, BMI, drinking, marital status, diabetes, education level, total cholesterol, uric acid, high sensitivity CRP or uric acid significantly modified the associations between quality and rapid eGFR_cr − cys_ decline (*P* > 0.05 for all).

## Discussion

To the best of our knowledge, the present prospective longitudinal study was the first study to demonstrate that poor sleep quality was associated with increased risk of CKD development in Chinese middle-aged and older people with normal kidney function, which provided clues to the risk factors affecting kidney function.

In recent years, the phenomenon of accelerated aging in China raises serious concerns for middle-aged and older people. As the circadian mechanisms becomes less efficient in the elderly, they tend to sleep less and have poor sleep quality, which may contribute to a series of health problems, such as cardiovascular diseases, depression, headache and memory loss (9). Some studies showed that poor sleep quality was associated with higher risk of coronary heart disease (24–26). In addition, higher proportion of depressive symptoms was associated with higher risk of rapid eGFR decline or CKD development in Chinese middle-aged or older adults with normal kidney function (27). Consistent with our study, another study from CHARLS suggested that long night-time sleep duration and poor sleep quality were associated with increased risk of CKD in middle-aged and older Chinese (18). This study highlighted the significant association between poor sleep duration and quality with the risks of CKD development in Chinese middle-aged or older people.

CKD were previously reported to be associated with night-time sleep duration and quality in middle-aged or older adults (18). However, previous studies demonstrated inconsistent results regarding the relationship between sleep duration and kidney function decline or CKD progression. An observed cohort study of 502,505 UK Biobank participants through clinical and genetic analyses indicated that either < 6 or ≥9 h of sleep duration was associated with a higher risk of CKD. Moreover, only short sleep duration was associated with the risk of end-stage kidney disease (ESKD) outcome when their study population was limited to males. While short sleep duration was associated with higher odds for CKD in genetic analysis (8). McMullan et al. (19) found that < 6 h sleep duration was significantly associated with higher risk of a faster eGFR decline in over 4,000 females. Moreover, a previous study of 3,600 Japanese workers indicated that short sleep duration (5 h/night) increased the risk of CKD in shift workers instead of non-shift workers, while long sleep duration was not a risk factor for CKD in shift and non-shift workers (28). Nevertheless, Nakajima et al. (29) showed that shorter sleep duration reduced the risk of CKD in Japanese males. The possible explanations for the inconsistency across different studies is probably because of the different reference groups used in the comparison and the different classifications of sleep duration, for example, 6–7 h (30), 6–8 h (14), and 7–8 h (29) were set as reference groups. In addition, < 6h, ≤ 5h and < 4h of nighttime sleep were regarded as short sleep duration in the different studies (14, 29, 30). Overall, the various studies showed that the relationships between sleep duration and the risk of kidney function decline remained unclear. In this study, neither night-time sleep duration nor daytime naps had any significant effect on rapid kidney function decline and the development of CKD in middle-aged and older adults with normal renal function. More studies are still needed to explore the reason for the inconsistencies.

In the older population, creatinine-based eGFR was inaccurate because diet, physical activity, and muscle mass could affect the creatinine levels (31). Cystatin Cis a cysteine protease inhibitor produced by nucleated cells. The serum cystatin C may vary due to insulin resistance or inflammation (32, 33). The kidney outcomes were assessed by eGFR_cr_ or eGFR_cys_ alone in previous studies (18, 34). Therefore, taking both cystatin C and creatinine measurement into consideration to determine the eGFR could improve the accuracy (35). The associations between sleep duration, quality and kidney outcomes defined by eGFR_cr − cys_ were not fully explored in previous studies. Some interesting findings were observed in our clinical analysis. The use of eGFR_cr − cys_ to evaluate kidney outcomes was more accurate compared to eGFR_cr_ or eGFR_cys_ alone (22, 36). The serum creatinine and cystatin C measurements across a longitudinal study (27) provided an opportunity to explore the associations between sleep duration, quality and rapid decline of kidney function among middle-aged or older population, which could be adjusted for known co-variables and stratified by various clinical characteristics. Multi-center sleep and CKD studies could be conducted to further investigate the findings of our study and to confirm the reciprocal relationship between them in the future.

Moreover, although analysis showed that no variables significantly modified the association between sleep quality and rapid eGFR decline (P interactions values > 0.05 for all), While BMI < 24 kg/m2, non-smoking, non-diabetic, married female participants strongly affected the associations between sleep quality and kidney function. However, given the multiple testing and similar directionality of most associations, these results may not have significant clinical impact. Meanwhile, the ability to detect moderate interactions was limited in the current sample size and larger number of samples are needed to verify the lack of influence by these variables in the future.

The mechanisms underlying the relationship between sleep and renal function need to be investigated further. We speculated that several potential mechanisms may participate in sleep duration and quality which affect renal function. Firstly, growing evidence showed that sleep duration was associated with the upregulation of inflammatory markers such as IL-6,TNF-a,CRP,AP-1 and STAT protein families which may activate immune response, aggravate kidney fibrosis and accelerate the decline of kidney function (37–44). However, there were no significant differences in high-sensitivity CRP levels between the different groups with different sleep durations and restlessness. Secondly, poor quality of nighttime sleep may disrupt circadian rhythms, which cause changes in serum hormone levels, insulin resistance, inverted cortisol rhythms and increased blood pressure (45, 46). These showed that sleep duration and quality were modifiable determinants of these established CKD risk factors (47–49). However, adjusting for blood pressure, glucose or self-reported heart disease did not affect the estimates for the risk of rapid renal function decline or developing to CKD in relation to sleep duration and quality. This suggested that either short sleep duration or quality are associated with rapid decline of renal function or progression to CKD *via* mechanisms independent of these known risk factors; or these endpoints did not eventually capture the vascular and metabolic consequences related to alterations in sleep duration and quality. Overall, these findings need to be verified, and their mechanisms investigated in the further studies.

This study has several limitations. Firstly, sleep duration and quality were self-reported, which may cause recall bias of sleep duration and quality. Self-reported sleep duration and quality is different from objectively measured sleep. In a study of 669 individuals, those with objective sleep measured as 5 h per night may overestimate their sleep duration by 1.3 h. While those participants with objective sleep measured as 7 h per night self-reported their sleep duration accurately (50). Secondly, the measurements of eGFR were only assessed at baseline and at the exit visit. If eGFR was changed due to other factors, the decline of eGFR from 2011 to 2015 would not accurately reflect the underlying change of eGFR during that period. More frequent measures of eGFR would improve accuracy for evaluating the progression of CKD over time. Thirdly, urine albumin was not measured at baseline in our cohort so neither adjustment for albuminuria nor analysis of the influence of urinary albumin secretion on the relationship between sleep duration, quality and kidney function could be performed. Fourthly, all of our study participants aged 45 years old and above were from China. Thus, it is unclear whether these findings can be applied to younger individuals or other ethnic groups. Fifthly, the number of participants whose sleep duration were >8 h was limited, so we were unable to assess whether there were associations between sleep duration and the decline of creatinine or cystatin C based on eGFR. Finally, in this observational study, some of the covariates used in the analyses were self-reported values. Hence, we are unable to rule out the possibility that our findings were confounded by unidentified factors.

## Conclusions

In summary, our analysis demonstrated that poor sleep quality was significantly associated with progression to CKD among Chinese middle-aged or older adults with normal kidney function. These findings paved the way for finding evidence toward potential therapeutic interventions to improve primary prevention of CKD.

## Data availability statement

Publicly available datasets were analyzed in this study. This data can be found here: http://charls.pku.edu.cn/, The China Health and Retirement Longitudinal Study (CHARLS) database.

## Ethics statement

The studies involving human participants were reviewed and approved by the Biomedical Ethics Review Committee of Peking University. The patients/participants provided their written informed consent to participate in this study.

## Author contributions

LZ and SX designed the study. LZ, SX, QD, FZ, YW, JJ, CG, JG, ML, and HZ analyzed the data. SX, YY, HJ, HX, SC, and HZ made the figures. ZH, SX, FZ, and JJ drafted and revised the paper. All authors approved the final version of the manuscript.
