# Supplementary material for: Association between sleep duration and quality with rapid kidney function decline and development of chronic kidney diseases in adults with normal kidney function: The China health and retirement longitudinal study
Source: Front Public Health. 2023 Jan 18;10:1072238. doi: 10.3389/fpubh.2022.1072238 (PMC9891205; doi:10.3389/fpubh.2022.1072238)
Supplement: Supplementary file 1 [file Data_Sheet_1.PDF]

Supplementary materials

Supplemental Figure 1. Flow chart of the participants in the study.

Supplemental Table 1. Characteristics of the included and excluded participants.

Supplemental Table 2. The association between night sleep duration and CKD outcomes defined by creatinine-based eGFR (eGFRcr-cys).

Supplemental Table 3. The association between daytime nap and CKD outcomes defined by creatinine-based eGFR (eGFRcr-cys).

Supplemental Table 4. The association between night sleep duration and CKD outcomes defined by creatinine-based eGFR (eGFRcr).

Supplemental Table 5. The association between daytime nap and CKD outcomes defined by creatinine-based eGFR (eGFRcr).

Supplemental Table 6. The association between sleep quality and CKD outcomes defined by creatinine-based eGFR (eGFRcr).

Supplemental Table 7. The association between night sleep duration and CKD outcomes defined by cystatin C-based eGFR (eGFRcys).

Supplemental Table 8. The association between daytime nap and CKD outcomes defined by cystatin C-based eGFR (eGFRcys).

Supplemental Table 9. The association between sleep quality and CKD outcomes defined by cystatin C-based eGFR (eGFRcys).

Supplemental table 10. Age stratified analyses for the association between sleep quality and CKD outcomes

Supplemental table 11. BMI stratified analyses for the association between sleep quality and CKD outcomes

Supplemental table 12. Total cholesterol stratified analyses for the association between sleep quality and CKD outcomes

Supplemental table13. Triglycerides stratified analyses for the association between sleep quality and CKD outcomes

Participants with blood data in the CHARLS 2011 (n=11847)

Exclude:
Missing baseline eGFR (n=2988)
Without information on sleep duration and quality (n=561)
Aged below 45 years old or without age information (n=137)

Participants with age ≥ 45 years old (n=8161)

Exclude:
With baseline eGFR< 60ml/min/1.73m2 (n=1117)

Participants with baseline eGFR ≥60ml/min/1.73m2 (n=7044)

Exclude:
Missing exit eGFR (n=2383)
Missing other demographic、clinical or biochemical information (n=575)

Participants enrolled in the final analysis (n=4086)

Supplemental Figure 1. Flow chart of the participants in the study.

Supplemental Table 1. Characteristics of the included and excluded participants

Variables	Included	Excluded	P-value
N	4086	7761	
Age, year	58.9 ± 8.7	59.2 ±10.3	0.121
Male, no. (%)	1755 (43.0)	3741 (48.2)	<**0.001**
Body mass index, kg/m2	23.7 ± 3.9	23.4 ± 4.0	**0.005**
Rural residence, no. (%)	3419 (83.7)	5898 (75.9)	<**0.001**
Married, no. (%)	3618 (88.5)	6784 (87.4)	0.073
Smoking status, no. (%)	1170 (28.6)	2443 (28.9)	<**0.001**
Drinking status, no. (%)	1282 (31.4)	1951 (25.1)	<**0.001**
Systolic BP, mmHg	130.2 ± 21.4	131.3 ± 22.2	**0.011**
Diastolic BP, mmHg	75.6 ± 10.9	76.0 ± 12.3	0.104
Diabetes, no. (%)	326 (8.0)	626 (8.1)	0.868
Self-reported heart disease, no. (%)	500 (12.2)	948 (12.2)	0.972
**Education, no. (%)**			<**0.001**
Illiteracy	1207 (29.5)	2189 (28.2)	
Literate	779 (19.1)	1411 (18.2)	
Primary school	936 (22.9)	1653 (21.3)	
Middle school or above	1164 (29.5)	2508 (33.2)	
**Laboratory results**			
Total cholesterol, mg/dl	193.5 ± 37.9	192.6 ± 39.0	0.218
Triglycerides, mg/dl	131.7 ± 90.8	136.7 ± 119.4	<**0.001**
HDL cholesterol, mg/dl	51.0 ± 15.1	51.7 ± 15.4	0.255
LDL cholesterol, mg/dl	116.6 ± 34.9	115.7 ± 34.9	0.180
Glucose, mg/dl	109.6 ± 34.2	110.7 ± 38.9	0.119
Hemoglobin A1c, %	5.3 ± 0.8	5.3 ± 0.8	0.863
eGFRcr-cys, ml/min per 1.73 m2	86.2 ± 14.4	75.4 ± 20.8	<**0.001**
Uric acid, mg/dl	4.3 ± 1.1	4.6 ± 1.3	<**0.001**
High-sensitivity CRP, mg/L	2.3 ± 5.5	1.1 ± 0.4	<**0.001**

Variables are presented as mean ± standard deviation or numbers (percentages).
p Values were calculated with two-sample t tests for continuous variables and chi-squared test for categorical variables.
BP, blood pressure; HDL Cholesterol, high density lipoprotein cholesterol; LDL Cholesterol, low density lipoprotein cholesterol; eGFRcr-cys, eGFR on the basis of a combination of serum creatinine and cystatin C; CRP, C-reactive protein.

Supplemental Table 2. The association between night sleep duration and CKD outcomes

CKD outcomes	Sleep duration (hours/night)	Events/N (%)	Odds Ratio (95% CI) P-value			
			Model 1		Model 2	
**Rapid decline in kidney function**						
	＜6	78/1228 (6.4%)	1.37 (1.02 - 1.84)	**0.037**	1.18 (0.86 - 1.63)	0.300
	6-8	136/2531 (5.4%)	Reference		Reference	
	＞8	30/327 (9.2%)	1.62 (1.05 - 2.50)	**0.028**	1.51 (0.95 - 2.38)	0.081
**Progression to CKD**						
	＜6	42/1228 (3.4%)	1.74 (1.15 - 2.64)	**0.009**	1.51 (0.98 - 2.33)	0.060
	6-8	50/2531 (2.0%)	Reference		Reference	
	＞8	10/327 (3.1%)	1.58 (0.79 - 3.15)	0.194	1.46 (0.73 - 2.96)	0.288

Model 1 was adjusted for eGFRcr-cys at baseline.
Model 2 was adjusted for age, gender, body mass index, smoking status, drinking status, systolic BP, diastolic BP, self-reported heart disease, glucose, total cholesterol, triglycerides, HDL cholesterol, eGFRcr-cys, uric acid.
BP, blood pressure; HDL Cholesterol, high density lipoprotein cholesterol; eGFRcr-cys, eGFR on the basis of a combination of serum creatinine and cystatin C; CKD, chronic kidney disease; CI, confidence interval.

Supplemental Table 3. The relationships between daytime nap and CKD outcomes

CKD outcomes	Daytime nap (hours/days)	Events/N(%)	Odds Ratio (95% CI) P-value			
			Model 1		Model 2	
**Rapid decline in kidney function**						
	0	131/1882 (7.0%)	0.80 (0.59 - 1.09)	0.161	0.83 (0.60 - 1.15)	0.263
	＜0.5h	923/388 (5.9%)	0.67 (0.39 - 1.16)	0.149	0.65 (0.37 - 1.15)	0.137
	0.5-1.5h	59/1244 (4.7%)	Reference		Reference	
	＞1.5h	31/572 (5.4%)	1.01 (0.68 - 1.52)	0.950	0.96 (0.62 - 1.48)	0.848
**Progression to CKD**						
	0	53/1882 (2.8%)	0.71 (0.46 - 1.10)	0.121	0.69 (0.44 - 1.09)	0.113
	＜0.5h	7/388 (1.8%)	0.55 (0.25 - 1.24)	0.150	0.59 (0.26 - 1.33)	0.203
	0.5-1.5h	26/1244 (2.1%)	Reference		Reference	
	＞1.5h	16/572 (2.8%)	0.65 (0.34 - 1.25)	0.193	0.62 (0.32 - 1.21)	0.163

Model 1 was adjusted for eGFRcr-cys at baseline.
Model 2 was adjusted for age, gender, body mass index, smoking status, drinking status, systolic BP, diastolic BP, self-reported heart disease, glucose, total cholesterol, triglycerides, HDL cholesterol, eGFRcr-cys, uric acid.
BP, blood pressure; HDL Cholesterol, high density lipoprotein cholesterol; eGFRcr-cys, eGFR on the basis of a combination of serum creatinine and cystatin C; CKD, chronic kidney disease; CI, confidence interval.

Supplemental Table 4. The association between night sleep duration and CKD outcomes

CKD outcomes	Sleep duration (hours/night)	Events/N(%)	Odds Ratio (95% CI)	P-value		
			Model 1		Model 2	
**Rapid decline in kidney function**						
	<6	144/1784 (8.1%)	1.30 (1.05 - 1.62)	**0.015**	1.20 (0.96 - 1.49)	0.107
	6-8	244/3736 (6.5%)	Reference		Reference	
	>8	41/471 (8.7%)	1.36 (0.96 - 1.93)	0.080	1.30 (0.91 - 1.84)	0.144
**Progression to CKD**						
	<6	61/1784 (3.4%)	1.30 (0.94 - 1.81)	0.116	1.21 (0.87 - 1.70)	0.262
	6-8	94/3736 (2.5%)	Reference		Reference	
	>8	16/471 (3.4%)	1.37 (0.80 - 2.35)	0.258	1.22 (0.71 - 2.11)	0.472

Model 1 was adjusted for eGFRcr at baseline.
Model 2 was adjusted for age, gender, body mass index, smoking status, drinking status, systolic BP, diastolic BP, self-reported heart disease, glucose, total cholesterol, triglycerides, HDL cholesterol, eGFRcr, uric acid.
BP, blood pressure; HDL Cholesterol, high density lipoprotein cholesterol; eGFRcr, eGFR on the basis of serum creatinine; CKD, chronic kidney disease; CI, confidence interval.

Supplemental table 5. The relationships between daytime nap and CKD outcomes

CKD outcomes	Daytime nap (hours/days)	Events/N(%)	Odds Ratio (95% CI)	P-value		
			Model 1		Model 2	
**Rapid decline in kidney function**						
	0	210/2789 (7.5%)	1.07 (0.85 - 1.33)	0.582	1.07 (0.85 - 1.35)	0.548
	＜0.5h	44/581 (7.6%)	1.06 (0.74 - 1.51)	0.743	1.05 (0.74 - 1.51)	0.779
	0.5-1.5h	136/1938 (7.0%)	Reference		Reference	
	＞1.5h	39/683 (5.7%)	0.78 (0.54 - 1.13)	0.187	0.80 (0.56 - 1.17)	0.249
**Progression to CKD**						
	0	89/2789 (3.2%)	1.14 (0.81 - 1.60)	0.457	1.15 (0.81 - 1.62)	0.439
	＜0.5h	14/581 (2.4%)	0.87 (0.48 - 1.57)	0.640	0.85 (0.47 - 1.55)	0.599
	0.5-1.5h	55/1938 (2.8%)	Reference		Reference	
	＞1.5h	13/683 (1.9%)	0.69 (0.37 - 1.27)	0.236	0.72 (0.39 - 1.32)	0.284

Model 1 was adjusted for eGFRcr at baseline.
Model 2 was adjusted for age, gender, body mass index, smoking status, drinking status, systolic BP, diastolic BP, self-reported heart disease, glucose, total cholesterol, triglycerides, HDL cholesterol, eGFRcr, uric acid.
BP, blood pressure; HDL Cholesterol, high density lipoprotein cholesterol; eGFRcr, eGFR on the basis of serum creatinine; CKD, chronic kidney disease; CI, confidence interval.

Supplemental table 6. The association between sleep quality and CKD outcomes

CKD outcomes	Restless sleep (days/week)	Events/N(%)	Odds Ratio (95% CI)	P-value		
			Model 1		Model 2	
**Rapid decline in kidney function**						
	0 - 2	260/3874 (6.7%)	Reference		Reference	
	3 - 7	169/2117 (8.0%)	1.22 (1.00 - 1.50)	0.052	1.20 (0.98 - 1.48)	0.079
**Progression to CKD**						
	0 - 2	93/3874 (2.4%)	Reference		Reference	
	3 - 7	78/2117 (3.7%)	1.52 (1.12 - 2.06)	**0.008**	1.50 (1.10 - 2.06)	**0.011**

Model 1 was adjusted for eGFRcr at baseline.
Model 2 was adjusted for age, gender, body mass index, smoking status, drinking status, systolic BP, diastolic BP, self-reported heart disease, glucose, total cholesterol, triglycerides, HDL cholesterol, eGFRcr, uric acid.
BP, blood pressure; HDL Cholesterol, high density lipoprotein cholesterol; eGFRcr, eGFR on the basis of serum creatinine ; CKD, chronic kidney disease; CI, confidence interval.

Supplemental Table 7. The association between night sleep duration and CKD outcomes

CKD outcomes	Sleep duration (hours/night)	Events/N(%)	Odds Ratio (95% CI)	P-value		
			Model 1		Model 2	
**Rapid decline in kidney function**						
	＜6	77/1110 (6.9%)	1.51 (1.10 - 2.07)	**0.011**	1.13 (0.79 - 1.62)	0.504
	6-8	132/2365 (5.6%)	Reference		Reference	
	＞8	26/304 (8.6%)	1.36 (0.846 - 2.22)	0.216	1.23 (0.71 - 2.11)	0.465
**Progression to CKD**						
	＜6	29/1110 (2.6%)	2.24 (1.33 - 3.79)	**0.003**	1.77 (1.02 - 3.09)	**0.043**
	6-8	28/2365 (1.2%)	Reference		Reference	
	＞8	5/304 (1.5%)	1.39 (0.53 - 3.64)	0.498	1.29 (0.48 - 3.45)	0.617

Model 1 was adjusted for eGFRcys at baseline.
Model 2 was adjusted for age, gender, body mass index, smoking status, drinking status, systolic BP, diastolic BP, self-reported heart disease, glucose, total cholesterol, triglycerides, HDL cholesterol, eGFRcys, uric acid.
BP, blood pressure; HDL Cholesterol, high density lipoprotein cholesterol; eGFRcys, eGFR on the basis of serum cystatin C; CKD, chronic kidney disease; CI, confidence interval.

Supplemental table 8. The relationships between daytime nap and CKD outcomes

CKD outcomes	Daytime nap (hours/days)	Events/N(%)	Odds Ratio (95% CI) Model 1	P-value Model 1	Odds Ratio (95% CI) Model 2	P-value Model 2
**Rapid decline in kidney function**						
	0	107/1762 (6.1%)	0.96 (0.69 - 1.33)	0.798	1.11 (0.77 - 1.60)	0.587
	＜0.5h	16/359 (5.3%)	0.71 (0.39 - 1.27)	0.248	0.69 (0.36 - 1.32)	0.261
	0.5-1.5h	81/1213 (6.7%)	Reference		Reference	
	＞1.5h	31/445 (7.0%)	1.06 (0.66 - 1.69)	0.814	1.08 (0.64 - 1.80)	0.780
**Progression to CKD**						
	0	27/1762 (3.2%)	0.81 (0.46 - 1.41)	0.449	0.90 (0.50 - 1.64)	0.740
	＜0.5h	6/359 (1.7%)	0.88 (0.36 - 2.18)	0.780	1.03 (0.40 - 2.64)	0.946
	0.5-1.5h	23/1213 (1.9%)	Reference		Reference	
	＞1.5h	6/445 (1.3%)	0.71 (0.29 - 1.75)	0.453	0.58 (0.22 - 1.50)	0.261

Model 1 was adjusted for eGFRcys at baseline.
Model 2 was adjusted for age, gender, body mass index, smoking status, drinking status, systolic BP, diastolic BP, self-reported heart disease, glucose, total cholesterol, triglycerides, HDL cholesterol, eGFRcys, uric acid.
BP, blood pressure; HDL Cholesterol, high density lipoprotein cholesterol; eGFRcys, eGFR on the basis of serum cystatin C; CKD, chronic kidney disease; CI, confidence interval.

Supplemental table 9. The association between sleep quality and CKD outcomes

CKD outcomes	Restless sleep (days/week)	Events/N(%)	Odds Ratio (95% CI)	P-value		
			Model 1		Model 2	
**Rapid decline in kidney function**						
	0 - 2	192/3042 (6.3%)	Reference		Reference	
	3 - 7	43/737 (5.8%)	0.93 (0.65 - 1.35)	0.709	0.81 (0.54 - 1.24)	0.337
**Progression to CKD**						
	0 - 2	42/3042 (1.4%)	Reference		Reference	
	3 - 7	20/737 (2.7%)	1.99 (1.16 - 3.41)	**0.012**	1.91 (1.08 - 3.38)	**0.026**

Model 1 was adjusted for eGFRcys at baseline.
Model 2 was adjusted for age, gender, body mass index, smoking status, drinking status, systolic BP, diastolic BP, self-reported heart disease, glucose, total cholesterol, triglycerides, HDL cholesterol, eGFRcys, uric acid.
BP, blood pressure; HDL Cholesterol, high density lipoprotein cholesterol; eGFRcys, eGFR on the basis of serum cystatin C; CKD, chronic kidney disease; CI, confidence interval.

Supplemental table 10. Age stratified analyses for the association between sleep quality and CKD outcomes

CKD outcomes	Restless sleep (days/week)	Events/N(%)	Odds Ratio (95% CI) Model 1	P-value	Model 2	
**Rapid decline in kidney function** (Age, years ＜65)	0 - 2	110/1971 (5.6%)	Reference		Reference	
	3 - 7	59/1049 (5.6%)	1.06 (0.76 - 1.48)	0.74	1.15(0.80 - 1.64)	0.45
**Progression to CKD** (Age, years ＜65)	0 - 2	30/1971 (1.5%)	Reference		Reference	
	3 - 7	24/1049 (2.3%)	1.51 (0.88 - 2.60)	0.13	1.52 (0.87 - 2.65)	0.14
**Rapid decline in kidney function** （Age, years ≥65）	0 - 2	38/659 (5.8%)	Reference		Reference	
	3 - 7	37/407 (9.1%)	1.65 (1.01 - 2.70)	**0.046**	1.70 (1.00 - 2.91)	0.051
**Progression to CKD** （Age, years ≥65）	0 - 2	21/659 (3.2%)	Reference		Reference	
	3 - 7	27/407 (6.6%)	2.14 (1.19 - 3.85)	0.11	2.33 (1.24 - 4.35)	**0.008**

Model 1 was adjusted for eGFRcr-cys at baseline.
Model 2 was adjusted for age, gender, body mass index, smoking status, rural residence, systolic BP, diastolic BP, self-reported heart disease, glucose, total cholesterol, triglycerides, HDL cholesterol, eGFRcr-cys, uric acid.
BP, blood pressure; HDL Cholesterol, high density lipoprotein cholesterol; eGFRcr-cys, eGFR on the basis of a combination of serum creatinine and cystatin C; CKD, chronic kidney disease; CI, confidence interval.

Supplemental table 11. BMI stratified analyses for the association between sleep quality and CKD outcomes

CKD outcomes	Restless sleep (days/week)	Events/N(%)	Odds Ratio (95% CI) P-value Model 1		Model 2	
**Rapid decline in kidney function**						
(Body mass index, kg/m2 ＜24)	0 - 2	76/1479 (5.1%)	Reference		Reference	
	3 - 7	55/891 (6.2%)	1.22 (0.85 - 1.76)	0.29	1.28(0.86 - 1.90)	0.22
**Progression to CKD**						
(Body mass index, kg/m2 ＜24)	0 - 2	26/1479 (1.8%)	Reference		Reference	
	3 - 7	31/891 (3.5%)	2.01 (1.19 - 3.41)	**0.009**	1.99 (1.16 - 3.42)	**0.013**
**Rapid decline in kidney function**						
(Body mass index, kg/m2 ≥24)	0 - 2	72/1151 (6.3%)	Reference		Reference	
	3 - 7	41/565 (7.3%)	1.28 (0.85 - 1.93)	0.24	1.45 (0.95 - 2.23)	0.085
**Progression to CKD**						
(Body mass index, kg/m2 ≥24)	0 - 2	25/1151 (2.2%)	Reference		Reference	
	3 - 7	20/565 (3.5%)	1.64 (0.90 - 2.98)	0.11	1.73 (0.94 - 3.21)	0.08

Model 1 was adjusted for eGFRcr-cys at baseline.
Model 2 was adjusted for age, gender, body mass index, smoking status, rural residence, systolic BP, diastolic BP, self-reported heart disease, glucose, total cholesterol, triglycerides, HDL cholesterol, eGFRcr-cys, uric acid.
BP, blood pressure; HDL Cholesterol, high density lipoprotein cholesterol; eGFRcr-cys, eGFR on the basis of a combination of serum creatinine and cystatin C; CKD, chronic kidney disease; CI, confidence interval.

Supplemental table 12. Total cholesterol stratified analyses for the association between sleep quality and CKD outcomes

CKD outcomes	Restless sleep (days/week)	Events/N(%)	Odds Ratio (95% CI)	P-value		
			Model 1		Model 2	
**Rapid decline in kidney function** (Total cholesterol, ＜200mg/dl)	0 - 2	78/1577 (4.9%)	Reference		Reference	
	3 - 7	51/861 (5.9%)	1.28 (0.88 - 1.85)	0.20	1.36(0.92 - 2.01)	0.13
**Progression to CKD** (Total cholesterol, ＜200mg/dl)	0 - 2	31/1577 (2.0%)	Reference		Reference	
	3 - 7	27/861 (3.1%)	1.63 (0.95 - 2.70)	0.08	1.53 (0.89 - 2.65)	0.12
**Rapid decline in kidney function** (Total cholesterol, ≥200mg/dl)	0 - 2	70/1053 (6.6%)	Reference		Reference	
	3 - 7	45/595 (7.6%)	1.18 (0.79 - 1.77)	0.43	1.26 (0.81 - 1.96)	0.31
**Progression to CKD** (Total cholesterol, ≥200mg/dl)	0 - 2	20/1053 (1.9%)	Reference		Reference	
	3 - 7	24/595 (4.0%)	2.17 (1.19 - 3.96)	**0.012**	2.44 (1.29 - 4.60)	**0.006**

Model 1 was adjusted for eGFRcr-cys at baseline.
Model 2 was adjusted for age, gender, body mass index, smoking status, drural residence, systolic BP, diastolic BP, self-reported heart disease, glucose, total cholesterol, triglycerides, HDL cholesterol, eGFRcr-cys, uric acid.
BP, blood pressure; HDL Cholesterol, high density lipoprotein cholesterol; eGFRcr-cys, eGFR on the basis of a combination of serum creatinine and cystatin C; CKD, chronic kidney disease; CI, confidence interval.

Supplemental table13. Triglycerides stratified analyses for the association between sleep quality and CKD outcomes

CKD outcomes	Restless sleep (days/week)	Events/N(%)	Odds Ratio (95% CI) Model 1	P-value	Model 2	P-value
**Rapid decline in kidney function** (Triglycerides, ＜150mg/dl)	0 - 2	76/1903 (4.0%)	Reference		Reference	
	3 - 7	55/1073 (5.1%)	1.26 (0.88 - 1.81)	0.20	1.28 (0.88 - 1.86)	0.20
**Progression to CKD** (Triglycerides, ＜150mg/dl)	0 - 2	35/1903 (1.8%)	Reference		Reference	
	3 - 7	34/1073 (3.2%)	1.74 (1.08 - 2.81)	**0.023**	1.73 (1.06 - 2.84)	**0.029**
**Rapid decline in kidney function** (Triglycerides, ≥150mg/dl)	0 - 2	72/727 (9.9%)	Reference		Reference	
	3 - 7	41/383 (10.7%)	1.36 (0.88 - 2.08)	0.17	1.50 (0.92 - 2.44)	0.10
**Progression to CKD** (Triglycerides, ≥150mg/dl)	0 - 2	16/727 (2.2%)	Reference		Reference	
	3 - 7	17/383 (4.4%)	2.18 (1.02 - 4.68)	0.045	1.97 (0.98 - 3.95)	0.056

Model 1 was adjusted for eGFRcr-cys at baseline.
Model 2 was adjusted for age, gender, body mass index, smoking status, rural residence, systolic BP, diastolic BP, self-reported heart disease, glucose, total cholesterol, triglycerides, HDL cholesterol, eGFRcr-cys, uric acid.
BP, blood pressure; HDL Cholesterol, high density lipoprotein cholesterol; eGFRcr-cys, eGFR on the basis of a combination of serum creatinine and cystatin C; CKD, chronic kidney disease; CI, confidence interval.
